# Bis(3,5-diiodo-2,4,6-trihydroxyphenyl)squaraine photodynamic therapy disrupts redox homeostasis and induce mitochondria-mediated apoptosis in human breast cancer cells

**DOI:** 10.1038/srep42126

**Published:** 2017-02-07

**Authors:** P. S. Saneesh Babu, Prasad M. Manu, T. Jayaram Dhanya, Pradhan Tapas, R. Nair Meera, Arun Surendran, Kumar A. Aneesh, S. Jisha Vadakkancheril, Danaboyina Ramaiah, S. Asha Nair, M. Radhakrishna Pillai

**Affiliations:** 1 Rajiv Gandhi Centre for Biotechnology, Cancer Research Program, Thiruvananthapuram, 695014, India; 2 CSIR-National Institute for Interdisciplinary Science and Technology, Photosciences and Photonics, Thiruvananthapuram, 695019, India; 3 Rajiv Gandhi Centre for Biotechnology Cardiovascular & Diabetes Disease Biology, Thiruvananthapuram, 695014, India; 4 CSIR-North East Institute of Science and Technology), Jorhat, 785006, India

## Abstract

Photodynamic therapy (PDT) is a clinically established and highly evolving treatment modality for cancer. PDT utilizes a light responsive drug called photosensitizer that selectively destroys tumor cells upon light irradiation. Squaraines are a class of dyes possessing all favorable characteristics of a photosensitizer and have been considered to be a potent candidate for next generation PDT. In this study we chose an iodo derivative of squaraine called diiodo-squaraine (bis(3, 5-diiodo-2,4,6-trihydroxyphenyl)squaraine) which has been reported for its tumor specificity but least studied for its cellular and molecular functions. Our studies revealed that the iodo derivative of squaraine possess maximum photodynamic activity in human breast cancer cells MDA- MB- 231 and had very little cytotoxicity in normal breast cells MCF-10A. We analyzed its pro and anti-apoptotic events initiated by oxidative stress exploring a proteomic approach and delineated other critical molecular pathways and key proteins involved in regulating the complex network of cellular response upon PDT. Our study showed that, diiodo- squaraines predominantly accumulate in mitochondria and induce mitochondria-mediated apoptosis. Our study also reveals the novel mechanistic role of diiodo-squaraines to induce oxidative stress there by activating both protective and death inducing pathways post PDT.

Breast cancer remains the leading cause of cancer death in women and the second most common cancer worldwide ensuing lung cancer^1^. The global burden of breast cancer exceeds all other cancers and the incidence rates of breast cancer are on the rise. Chest wall reccurence after mastectomy presents a major challenge in breast cancer treatment^2^. Surgical exclusion, radiotherapy, or together, are the collective treatment modality for chest wall metastasis^2^,^3^,^4^. Usual anticancer treatment modalities, like chemotherapy and radiotherapy are not tumor specific and patients suffer from severe side effects due to loss of healthy cells^5^. Hence development and study of new targeted therapeutic strategies for breast cancer treatment need to be prioritized.

PDT is one such treatment strategy which involves the administration of a compound called Photosensitizer (PS), which accumulates in the malignant cells, followed by targeted illumination of the tumor with a laser of appropriate wavelength. Subsequently, this results in a sequence of photochemical events that generate reactive oxygen species (ROS), triggering oxidative damage and eventually cell death^6^,^7^,^8^,^9^. An ideal photosensitizer for PDT should have absorption in the Near Infrared Region(NIR) (600–850 nm), wherein the tissue penetration by light is higher^10^. The sensitizer should be cytotoxic only in the presence of light with minimal dark toxicity and should be rapidly excreted from the body. Currently available photosensitizers have poor absorption spectrum in NIR region and show significant dark toxicity. To overcome these major hurdles associated with the existing photosensitizers, there has been great interest in the development of dyes that possess absorption photodynamic window^11^,^12^,^13^. Among these; the squaraine dyes have drawn immense interest in recent years.

Considering the efficacy of PDT and the extent of its applications, a range of second generation photosensitizers^14^,^15^,^16^, such as squaraines^17^,^18^,^19^ are now being evaluated for their compatibility in cancer therapy, and it is important to explicate their mechanisms of action in PDT. Squaraines being a class of dyes possessing sharp and intense absorption in the NIR region are reported to exhibit significant triplet and quantum yields. *In vitro* experiments on several squaraines illustrate them to be highly phototoxic and not photomutagenic^20^. Diiodo-squaraine (bis(3,5-diiodo-2,4,6-trihydroxyphenyl)squaraine) possess targeted accumulation in tumor cells rendering it suitable for selective destruction without affecting normal cells^21^,^22^.

Understanding the molecular mechanism behind diiodo-squaraine mediated PDT has been predicted to unravel novel pathways. There are two types of photochemical reactions, Type I involves an electron transfer between biological molecules and excited photosenisitizer, whereas Type II involves direct energy transfer from excited photosensitizer to the surrounding molecular oxygen and generation of singlet oxygen. However, both these pathways ultimately induce oxidative stress in cells. PDT predominantly induces ROS mediated cell death pathways via metabolic byproducts like hydrogen peroxide, hydroxyl radical and superoxide anion^23^. Typically ROS levels are very high in cancer cells when compared to normal cells attributed to their higher metabolic rate; therefore, minor increase in ROS levels usually trigger various cell death pathways. In normal cells, ROS are produced at low concentrations and therefore are effectively neutralized by potent antioxidant mechanisms within the cell^24^. So it is very crucial to understand the mechanistic role of diiodo-squaraine induced cell death, which led us to investigate various molecular events initiated by oxidative stress induced by diiodo-squaraine based PDT.

Human breast cancer cells MDA-MB-231 was employed for all molecular analysis since we observed highest photodynamic activity of diiodo-squaraine in MDA-MB-231 cells. The molecular mechanism behind PDT mediated cell death greatly depends upon its subcellular localization because the locale of action could be highly restricted to a radius of about 40 nm^25^,^26^. Subcellular localization of a photosensitizer in extremely vulnerable cell organelles would probably enhance its efficacy up to 3000 times, when compared to a photosensitizer localized in extracellular matrix or cell membrane^27^. Hence, it remained as our priority to investigate the intracellular localization of this diiodo-squaraine.

To explore the cellular response to diiodo-squaraine based PDT, we performed a proteome profiling using LC-MS/MS. Tryptically digested extracts of MDA-MB-231 cells followed by PDT with diiodo-squaraine were analyzed by a data independent acquisition workflow (LC-MS/MS) in three technical replicates. Pathway analysis was done using DAVID Bioinformatics Resources^28^. Validation of the identified pathways was examined using Real time PCR, western blotting, flow cytometry and fluorescent microscopic analysis. In the present study, we report that diiodo-squaraine induces oxidative stress mediated cell death pathways.

## Results

### Diiodo-squaraine shows cytotoxicity predominantly in cancer cells

**Figure 1:**
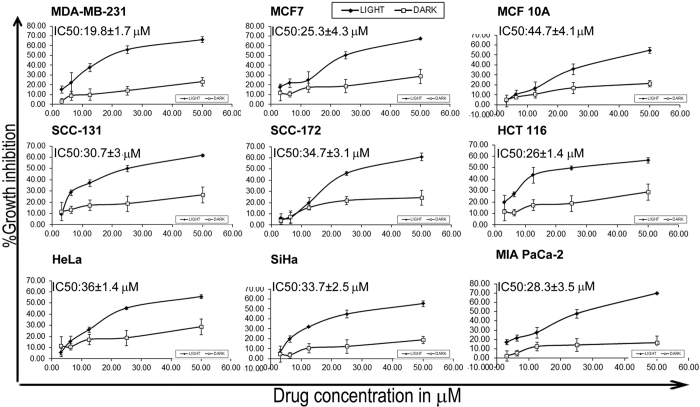
MTT assay of diiodo-squaraine in a panel of cell lines. MTT assay was done on a panel of cancer cells and shows cytotoxicity of diiodo-squaraine in the presence and absence of light. Data are expressed as a mean value ± standard deviation of three independent experiments. Diiodo-squaraine shows significant cytotoxicity in the presence of light in all cells, butshows negligible cytotoxicity in the absence of light. Here we observed maximum cytotoxicity in breast cancer cell MDA-MB-231 with an IC_50_ value of 19.8 ± 1.7 μM and minimum cytotoxicity in normal breast cells MCF 10A.

The cytotoxicity of diiodo-squaraine was evaluated in nine cell lines under light and dark condition, as tabulated in Fig. 1. Cytotoxicity screening of the diiodo-squaraine was carried out in eight cancer cell lines to identify the cell line exhibiting highest cytotoxicity. Cytotoxic analyses have shown that diiodo-squaraine is fundamentally non-cytotoxic in dark, but remarkably exhibits high photo-cytotoxicity. The IC_50_ value observed in breast cancer cells MDA-MB-231 cells (19.8 ± 1.7 μM) and MCF 7 (25.3 ± .4.3 μM) cells was about two fold lower than those for normal immortalized breast cells-MCF 10 A (44.7 ± 4.1 μM). The IC_50_ value of SCC-131 and SCC-172 (Oral cancer cells) was 30.7 ± 33 μMand 34.7 ± 3.1 μM respectively. HeLa and SiHa (cervical cancer cells) exhibited IC_50_values of 36 ± 1.4 μM and 33.7 ± 2.5 μM respectively. IC_50_ values for pancreatic cancer MIA PaCa-2 cells and colon cancer HCT 116 cell was 28.3 ± 3.54 μM and 26.1 ± 1.4 μM respectively. This study showed that MCF 10 A and MDA-MB-231showed minimal and maximal cytotoxicity respectively. Therefore all further molecular analysis of diiodo-squaraine PDT was carried out in MDA-MB-231.

### Diiodo-squaraine induced ROS generation in MDA-MB-231 cells

**Figure 2:**
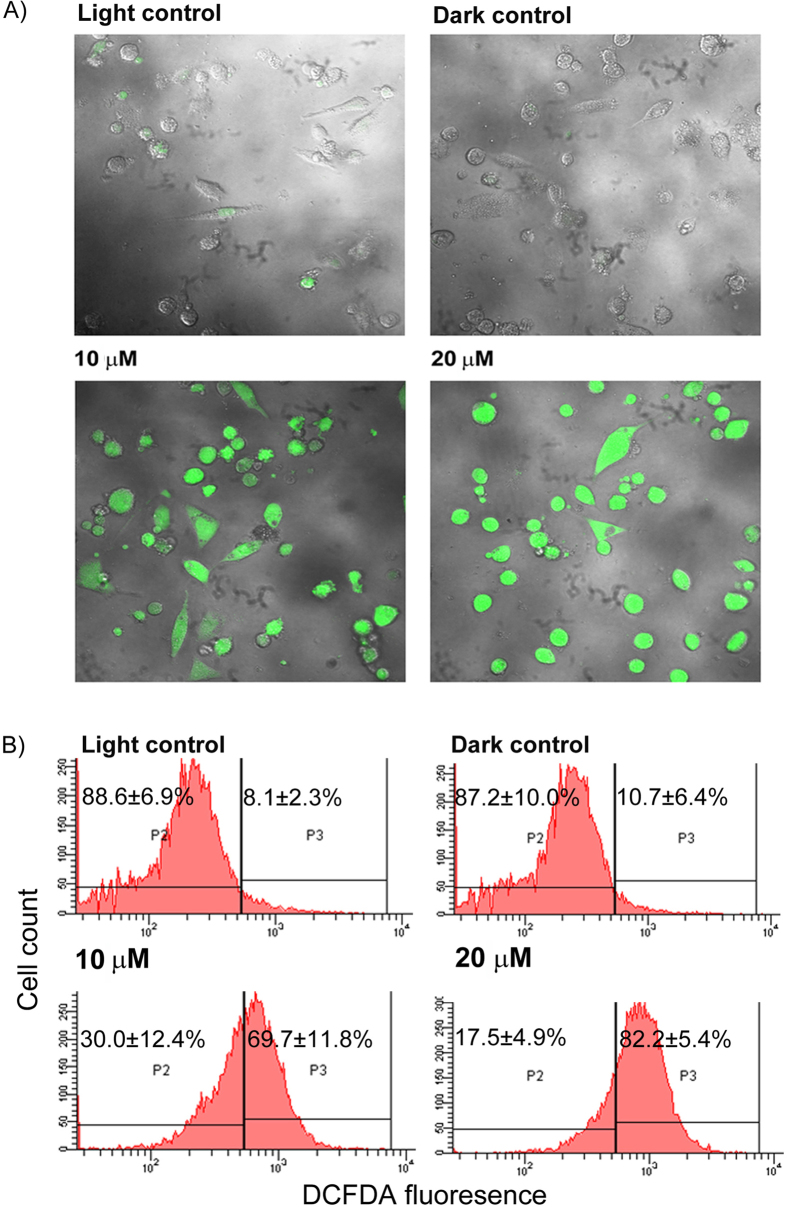
Sub cellular ROS induction after PDT with diiodo-squaraine. CM-H2DCFDA showed enhanced ROS levels after PDT with squaraine in MDA-MB-231 cells. (**A**) Confocal images of MDA-MB-231 after PDT with diiodo-squaraine, cells shows enhanced fluorescence of CH-H2DCFDA which is directly proportional to cellular ROS (**B**) Flow cytometric analysis of MDA-MB-231 cells, showing induction of intracellular ROS by squaraine in PDT. Data are expressed as a mean value ± standard deviation of three independent experiments. Here the population P2 shows background fluorescence, which represents cells with low ROS, and the population P3 shows cells with enhanced fluorescence indicating cells with high ROS. Here we can observe a significant increase P3 population both at 10 and 20 μM diiodo-squaraine in PDT when compared to both the controls.

The anticancer activity of a photosensitizer depends on the ROS mediated adaptive and cell death responses^29^. Hence we studied the cellular ROS stress upon diiodo-squaraine treatment using the CMH_2_DCFDA probe. Confocal microscopy with CMH_2_DCFDA showed enhanced green fluorescence implying high ROS stress which was further validated and quantified using flow cytometric analysis with the same probe. Hike in the ROS population from 8.1 ± 2.3% (Light control) & 10.7 ± 6.4% (Dark control) to 69.7 ± 11.8% (10 μM) and 62.2 ± 5.4% (20 μM) was observed upon PDT with diiodo-squaraine in MDA-MB-231 cells (Fig. 2). Thus, PDT with diiodo-squaraine induced a significant subcellular ROS generation leading to oxidative stress in MDA-MB-231 cells.

### Diiodo-squaraine induced unfolded protein response upon PDT

**Figure 3:**
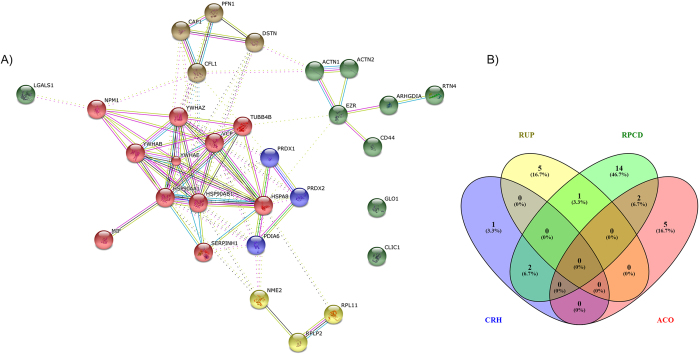
STRING Network analysis and Venn diagram of proteome analysis. Network interaction of up-regulated proteins (2 fold and above) in MDA-MB-231 Upon PDT with diiodo-squaraine (20 μM) compared to Dark control by STRING analysis. Here we observed overlap of proteins to Regulation of programmed cell death (RPCD) to other pathways.

**Figure 4:**
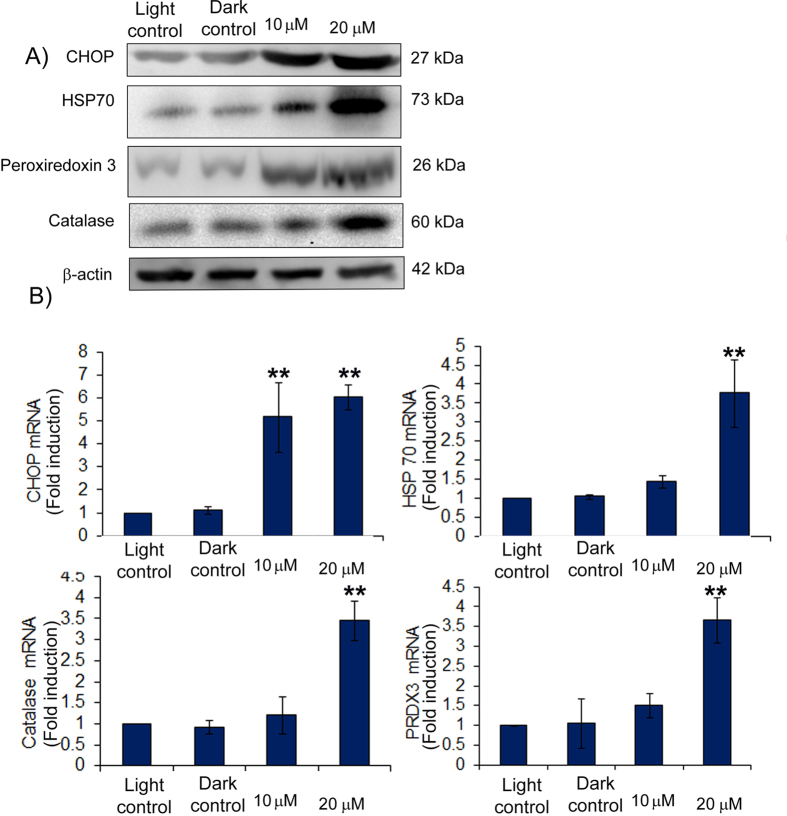
Western blotand real-time PCR results show simultaneous activation of redox signaling molecules and UPR signaling. (**A**) Western blot result showsupregulation of UPR associated proteins CHOP and HSP70 and Redox associated proteins Peroxiredoxin 3 and Catalase. (**B**) mRNA levels ofthe UPRgenes**i**n breast cancer cells MDA-MB-231 cells were analyzed by quantitative realtime PCR (expression was normalised to L19 RNA expression). Experiments were performed in triplicate. Data are expressed with a standard deviation from the mean (n = 3). Our results shows fold induction of UPR associated genes CHOP and HSP70 and redox associated genes PRDX3 and catalase.^**^P < 0.01, compared with the Light control.

Oxidative damage to proteins leads to protein unfolding followed by accumulation in the endoplasmic reticulum (ER) causing ER stress. To restore the function of ER, cells react by unfolded protein response (UPR). Protein profiling data revealed the upregulation of UPR proteins upon PDT with diiodo-squaraine in MDA-MB-231 (Supplementary Data; Table S1). Further studies with STRING network analysis depicts strong interaction between proteins involved in UPR and programmed cell death pathways (Fig. 3). Key proteins involved in ER stress such as CHOP (multifunctional transcription factor) and HSP70 (molecular chaperone and sensor of ER stress)^30^ were observed to be up-regulated at mRNA as well protein levels (Fig. 4) after PDT with diiodo-squaraine.

### Expression of cell redox homeostasis proteins augmented by diiodo-squaraine PDT in MDA-MB-231 cells

All living cells maintain redox environment by a biological process called cell redox homeostasis. Since diiodo-squaraine PDT promotes intense ROS, the escape mechanism from oxidative stress will be by activating redox signaling. Proteomic study ascertained the enrichment of cell redox proteins (Supplementary Data; Table S2) and STRING network analysis of the same revealed a strong link with programmed cell death pathway (Fig. 3). Among the observed proteins, peroxiredoxin 3 and catalase (redox homeostasis proteins) showed significant upregulation of mRNA with concomitant protein expression further confirming activation of redox signaling in diiodo-squaraine mediated PDT (Fig. 4).

### Diiodo-squaraine induced actin cytoskeletal disintegration followed by PDT

**Figure 5:**
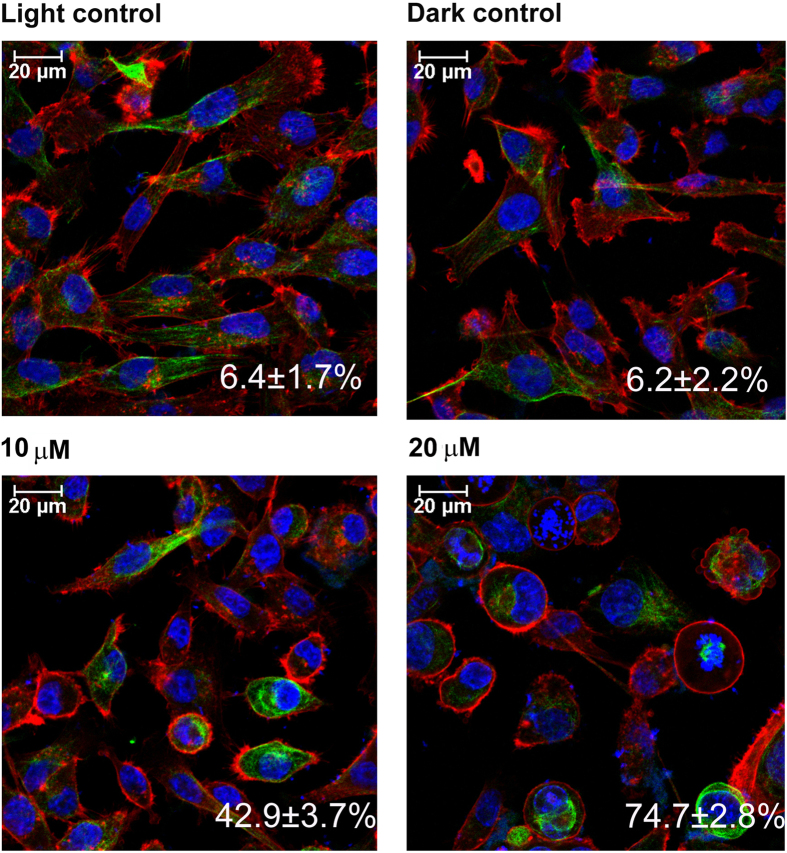
Immunocytochemistry showing cytoskeletal dynamics after PDT with diiodo-squaraine in MDA-MB-231. Actin stained with Phalloidin TRITC (Red) and Tubulin with anti α-Tubulin antibody and secondary FITC (green) and nucleus Hoechst (blue). Scale bars represent 20 μm. We observed the structural changes in actin cytoskeleton which was spindle shaped in both controls and less ring-shaped actin organisation of about 6.4 ± 1.7% in light control and 6.2 ± 2.2%in dark control. Upon PDT we observed more ring shaped actin about 42.9 ± 3.7% with 10 μM and 74.7 ± 2.8% with 20 μM diiodo-squaraine treatment.

Actin cytoskeleton plays a significant role in cell death and survival followed by oxidative stress^31^. We analyzed the involvement of cytoskeletal dynamics *via* DAVID data analysis of LC-MS/MS data followed by confocal imaging of actin cytoskeleton. Actin cytoskeletal organization signals were activated during diiodo-squaraine PDT by LC-MS/MS analysis (Supplementary Data; Table S3). Further interactions were observed by STRING network analysis (Fig. 3). Immunocytometric staining with Phalloidin–Tetramethyl rhodamine B isothiocyanate analysis of tubulin showed diiodo-squaraine at concentrations of 10 and 20 μM, on photo irradiation induced direct actin and tubulin condensation and formation of ring like structures, which suggested the involvement of cytoskeletal dynamics with apoptosis (Fig. 5).

### Diiodo-squaraine localized predominantly in the mitochondria and induced loss of mitochondrial membrane potential (∆ψM) following PDT

**Figure 6:**
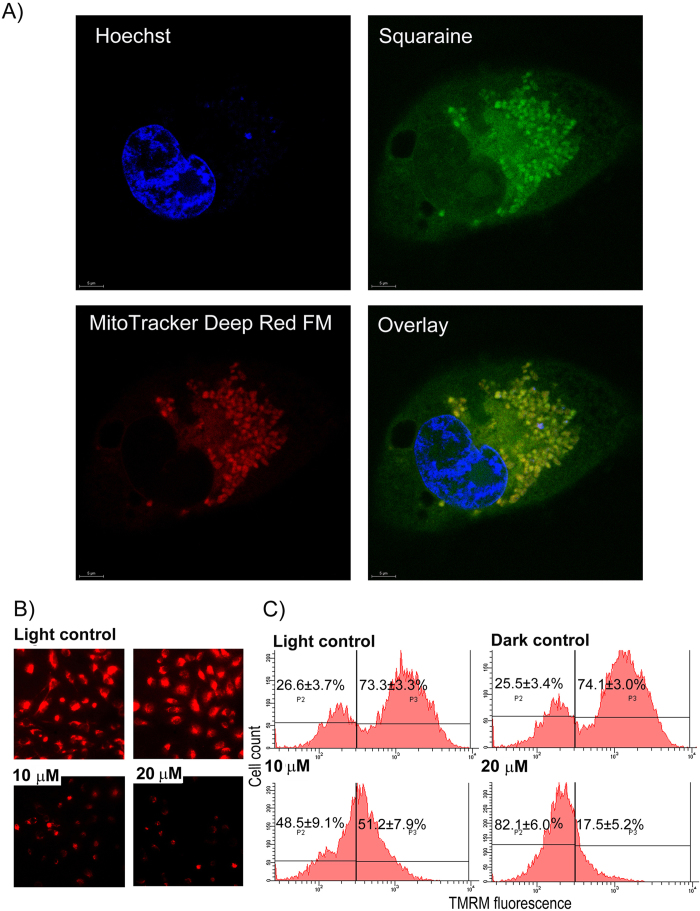
Diiodo-squaraine localizes to mitochondria and induces loss of mitochondrial membrane potential upon PDT. (**A**) MDA-MB-231 cells, where treated with 100 μM diiodo-squaraine for 1 hour followed by MitoTracker-far red and Hoechst counter staining shows diiodo-squaraine predominantly localize in mitochondria. Scale bars represent 5 μm (**B**)The changes in mitochondrial membrane potential were monitored using the Tetramethylrhodamine methyl ester (TMRM), which is a cell-permeant, cationic, red-orange fluorescent dye that is readily sequestered by active mitochondria. The red fluorescence of TMRM is directly proportional to Mitochondrial membrane potential (∆ψM). TMRM shows loss of ∆ψMs after PDT with diiodo-squaraine in MDA-MB-231 cells. a) Fluorescent microscopic images of MDA-MB-231 after PDT with diiodo-squaraine cells showsthe reduced fluorescence of TMRM which is directly proportional to ∆ψM **C**) Flow cytometric analysis of MDA-MB-231 cells, showing loss of ∆ψM by diiodo-squaraine in PDT. Data are expressed as a mean value ± standard deviation of three independent experiments. Here the population P2 shows background fluorescence, which represents cells with low ∆ψM, and the population P3 shows cells with enhanced fluorescence indicating cells with high ∆ψM. Here we can observe a significant decrease in P3population both at 10 and 20 μM diiodo-squaraine in PDT when compared to both controls.

To study the subcellular localization of photosensitizers for predicting its biological action, we used fluorescent imaging for the localization study. The subcellular photosensitizer distribution was analyzed by confocal laser scanning microscopy (Fig. 6A). We observed diiodo-squaraine to be predominantly localized in mitochondria. The changes in ∆ψM was monitored using tetramethylrhodamine methyl ester (TMRM), a cell-permeant, cationic, red-orange fluorescent dye that is readily sequestered by active mitochondria causing a decrease in red fluorescence on apoptosis. Our results showed time and concentration dependent changes in ∆ψM. We observed minor reduction of ∆ψM in sub-lethal dose of PDT (5 μM) and significant reduction in lethal dose (10 and 20 μM) 3 hours of post PDT. We also observed the reduction of ∆ψM in 6,12 and 24 hours of PDT, when compared to light and dark controls (Supplementary Figure SF 1 and Fig. 6). These findings confirmed that PDT with diiodo-squaraine could induce concentration dependent loss of ∆ψM at early time points.

### Diiodo-squaraine PDT showed time and concentration dependent apoptosis

**Figure 7:**
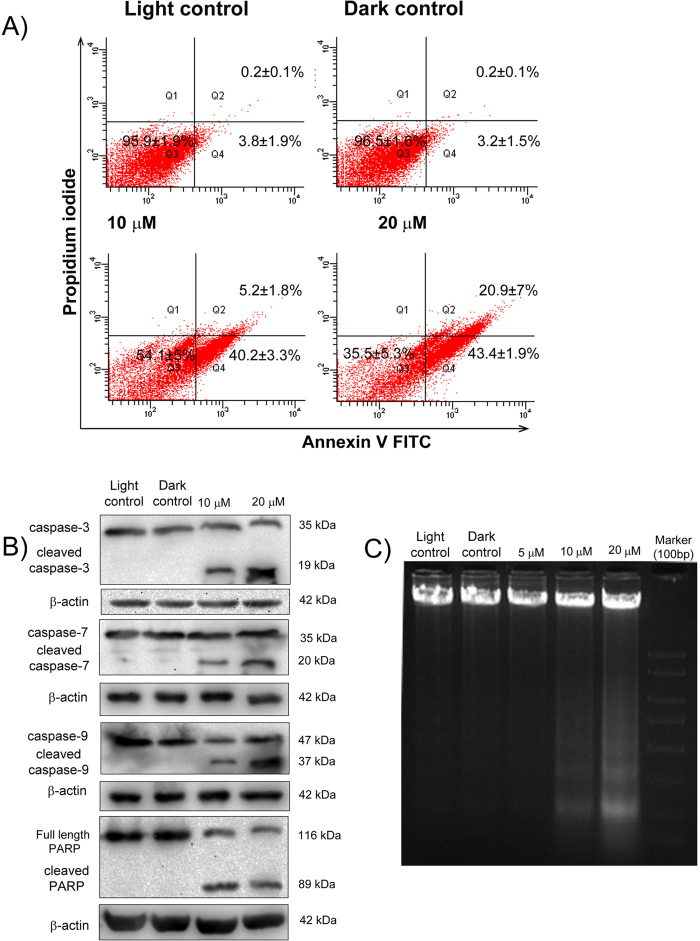
Apoptotic induction of diiodo-squaraine in PDT. (**A**) Annexin Flow cytometric analysis of MDA-MB-231 cells after PDT treatment with light control and dark control and in presence of diiodo-squaraine at concentration 10 μM and 20 μM. Data are expressed as a mean value ± standard deviation of three independent experiments. The lower left quadrant (Q3) of the panel shows the viable cells, negative for both Annexin V−FITC and PI. The lower right quadrants (Q4) represent the early apoptotic cells Annexin V−FITC and Propidium iodide (PI). The upper right quadrants (Q2) represent the late apoptotic cells positive for Annexin V−FITC and Propidium iodide (PI). Our results shows that cells undergoing early and late apoptosis on diiodo-squaraine PDT was exclusively concentration dependent (**B**) Western blot analysis shows activation of caspase 3, 7, 9 followed by PARP cleavage (**C**) DNA ladder formation using 10 and 20 μM diiodo-squaraine PDT confirms that DNA fragmentation was concentration dependent.

To understand the mechanism behind PDT activity and cellular damage induced by diiodo-squaraine, we investigated the apoptotic potential of diiodo-squaraine using Annexin V-FITC/PI flow cytometric analysis as shown in Fig. 4. The cell populations at different phases of cell death, namely, viable (AnnexinV-FITC (−ve)/PI (−ve)), early apoptotic (Annexin V-FITC (+ve)/PI (−ve)) and necrotic or late-stage apoptotic (Annexin V-FITC(+ve)/PI(+ve)) were examined using different drug concentration after 24 hours of PDT (Fig. 7A) and the results revealed that diiodo-squaraine PDT induced apoptosis in a concentration dependent manner. Time dependent study indicated cells to be viable at early time points (3 hours) in all concentrations (5, 10 and 20 μM). This clearly showed ∆ψM loss is the first event which gradually leads to cell death. We observed gradual increase in apoptotic population in 6 hours and 12 hours of PDT in lethal concentrations (10 and 20 μM) whereas in death in sub-lethal dose of PDT (5 μM) we didn’t observed significant cell (Supplementary Figure SF 2). Diiodo-squaraine of concentration10 μM induces 40.2 ± 3.3% early apoptosis and 5.2 ± 1.8% late apoptosis and at 20 μM 43.4 ± 1.9% early apoptosis and 20.9 ± 7% late apoptosis in 24 hours of post PDT (Fig. 7). Our results showed that cell death induced by PDT is both time and concentration dependent.

### Diiodo-squaraine PDT triggers caspase cascade and DNA fragmentation

Loss of ∆ψM usually leads to cytochrome c release and triggers a cascade of events eventually culminating in programmed cell death (PCD). So we analyzed whether caspases are involved in cell death induced by diiodo-squaraine. Western blot data showed that caspases involved in diiodo-squaraine mediated apoptosis in MDA- MB -231 cells are caspase 3 which resulted in the induction of caspase 7 and 9 (Fig. 7B). Cleaved PARP is an established marker for apoptosis^32^. We observed PARP cleavage at both the concentrations of 10 and 20 μm diiodo-squaraine in PDT, confirming the induction of apoptotic signaling pathway (Fig. 7B). It has also been observed that diiodo-squaraine induced a concentration dependent DNA fragmentation, which has been established as a hallmark of late-stage apoptosis (Fig. 7C).

### Diiodo-squaraine PDT induces cascade of molecular events which leads to apoptosis

Reactive oxygen species (ROS) and the resulting oxidative stress plays an essential role in apoptosis^33^. We found that PCD pathways had a significant role in eliciting the apoptotic pathway proteins (Supplementary Data; Table S4) Association with Regulation of Programed Cell Death (RPCD) to other observed pathways like Cell Redox Homeostasis (CRH), Response to Unfolded Protein (RUP) and Actin Cytoskeletal Organization (ACO) as observed in the VENN diagram (Fig. 3B) and STRING network analysis implicated that there lies a strong interaction between these pathways (Fig. 3A).

## Discussion

PDT has emerged as a potential anticancer therapeutic strategy already in clinics. We initiated our study using an emerging class of photosensitizer called diiodo-squaraine to validate its therapeutic efficacy by discerning the molecular mechanisms. Prior studies suggested that a diiodo variant of squaraine dye showed superior activity over other derivatives and accumulates selectively in cancer cells thereby rendering minimal toxicity to healthy cells^21^. Hence, we selected diiodo-squaraine as the candidate molecule to further pursue our study, since the site of action and mechanism of diiodo-squaraine PDT remained to be thoroughly elucidated. To analyze the cell line specificity of diiodo-squaraine we employed standard MTT assay in eight cancer cell lines and a normal cell line. Our preliminary cytotoxicity assay data authenticates the highest photodynamic efficiency of diiodo-squaraine in breast cancer cell MDA-MB-231, hence we selected this cell line for further studies. We observed that diiodo-squaraine showed higher activity in almost all cancer cells employed in our study (MDA-MB-231, MCF 7, SCC-131, SCC-172, MIA PaCa-2, HCT116, HeLa and SiHa) as compared to the normal breast immortalized cell line MCF 10 A (Fig. 1). Even though the molecular mechanisms warranted a detailed deliberation, we have attempted to reveal novel oxido-redox pathways activated by diiodo-squaraine PDT in this study.

We applied a proteomic approach to prospect the molecular basis of diiodo- squaraine in PDT which revealed the activation of molecular pathways like response to unfolded protein, cell redox homeostasis, regulation of programmed cell death and actin cytoskeletal organization. It has been widely accepted that cell death induced by PDT is highly dependent on oxidative stress mediated cell death pathways. Thus, the increased cytotoxicity in cancer cells exhibited by diiodo-squaraines might be due to their ROS generation as we observed high ROS induction in CMH2DCFDA assay on diiodo-squaraine treatment (Fig. 2). Redox regulation in cancer cells in response to oxidative stress has a key role in modern molecular therapeutics^34^. Modulation of redox signaling can induce cell death specific to cancer cells^35^. Cells respond to any oxidative stress by way of its redox mechanisms but beyond a threshold level these redox mechanism could initiate cell death, hence redox associated molecules has been reported as molecular markers for oxidative stress^36^,^37^,^38^,^39^.

Elevated level of antioxidant molecules in cancer cells can protect cells from cytotoxic effect of PDT^40^. Our proteomic profiling, immunoblots and real-time quantitative PCR data substantiate the activation of antioxidant molecules specifically catalase and peroxiredoxin 3. Our data depict apoptosis to ensue even under redox activation, when oxidative stress seemed to be above a threshold level. Various pro-apoptotic stimuli increase the production of ROS in mitochondria and the cells will try to protect themselves by modulating overexpression of ROS scavenging enzymes like catalase and peroxiredoxin^41^. The constitutive oxidative stress of cancer cells are very high, so further addition of ROS in cell will induce higher ROS threshold set point which cannot be scavenged and may lead to mitochondrial damage and release of caspase thus triggering apoptosis^42^. This might be the reason behind the differential cytotoxicity between normal (MCF 10A) and cancer cells treated by diiodo-squaraine. In clinical PDT scenario innermost cells in a tumor may receive insufficient light dose for exciting a photo sensitizer (due to scattering and reflection) therefore a chance to overcome the ROS stress induced by PDT via activation of ROS scavenger enzymes as observed in our study. So, further studies in sub lethal dose of diodo-squaraine PDT may give a better understanding of PDT escape mechanism by antioxidant genes.

Prolonged ROS stress in a cell could lead to oxidative damage to proteins followed by unfolding and accumulation in ER which later may lead to ER stress mediated apoptosis^43^. Our proteomic data demonstrated that, diiodo-squaraine induced activation of the pathways related to response to unfolded protein. The immunoblot and quantitative real time PCR data (Fig. 4) indicated CHOP as a key molecule which regulated ER stress mediated apoptosis, HSP70 a known biomarker for unfolded protein response, was also activated upon PDT with diiodo-squaraine.

It has been reported that ROS has a direct role in cytoskeletal dynamics^44^. The actin cytoskeleton have a significant role in apoptosis which involves changes in actin filament organization at different stages of apoptosis^45^. Manipulation of actin cytoskeletal dynamics has evolved as a therapeutic strategy to induce apoptosis^46^,^47^. The morphological changes in cell rounding, requires the formation of a contractile cortex of myosin II and special organization of actin filaments^48^. Withdrawal of the actin-myosin II cortex will change membrane dynamics triggering the creation of membrane blebs^48^,^49^. At the last steps of cell death the actin cytoskeleton is tainted and phagocytosis of the apoptotic bodies arises^49^. Here we observed circular formation of actin cytoskeleton (Fig. 5) and activation of pathways related to actin cytoskeletal organization as evidenced from our proteomic data profiling (Supplementary Data: Table S3).

The efficiency and mode of action of a photosensitizer depends on its intracellular localization. The action of a photosensitizer remain restricted to a radius of not more than 40 nm of its localization^25^. The confocal imaging studies revealed that diiodo-squaraine was predominantly localized in mitochondria (Fig. 6A) and these results supported the loss of ∆ψM post PDT (Fig. 6B and Supplementary Figure SF 1). Rapozzi *et al*.^50^ have studied the cellular localization and cell death mechanism of benzothiazole based Squaraines. They showed benzothiazole based Squaraines was distributed all over cytoplasm in HeLa cells and proposed necrosis as the primary cell death pathway. In contrast to that, we report that our compound diiodo-squaraine localized in mitochondrion and induced apoptotic cell death. Our study shows significant ∆ψM loss happening even in 3 hours and also in lethal dose (10 and 20 μM) of PDT (Supplementary Figure SF 1). However, all cells in early time periods (3 hours) were perceived to be healthy and apoptosis was discerned at 6 hours and 12 hours post PDT. This observation clearly conveys that, loss of ∆ψM triggers a cascade of signaling events which include, Caspase 3, 7, 9 activation, PARP cleavage the hall mark of apoptosis and DNA ladder formation.

**Figure 8:**
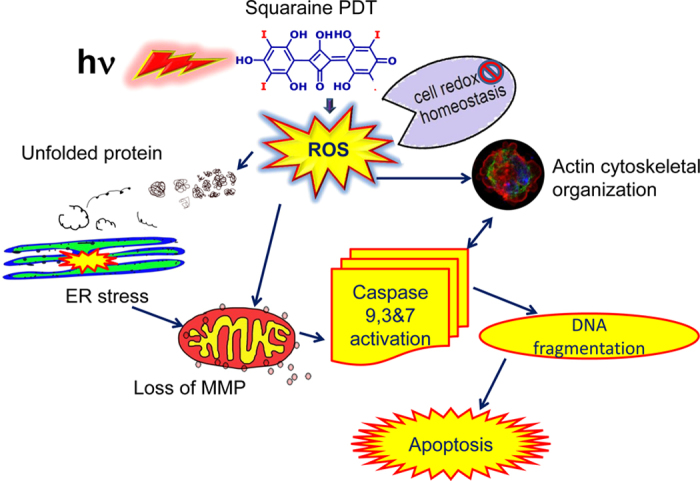
Proposed model of diiodo-squaraine induced cell death. We propose a mechanism of tumor cell killing by diiodo-squaraine -PDT. Upon PDT, diiodo-squaraine induces oxidative stress to the cells. Prolonged oxidative stress induces unfold protein response and Endoplasmic reticulum(ER) stress followed by loss of Mitochondrial membrane potential. On the other hand, induction of ROS can induce loss of mitochondrial membrane potential and caspase activation leads to DNA fragmentation and apoptosis. ROS induced by PDT also induces cell redox homeostasis which may protect the cells from oxidative damage and possibly induce PDT resistance. We observed changes in actin dynamics which was caused by either, via direct oxidative damage of actin cytoskeleton or indirectly *via* caspase cascade.

In summary, we observed that diiodo-squaraine showed the highest photodynamic efficiency in MDA-MB-231 (breast) compared to other cancer cells (oral, cervical, colon and pancreatic). We also demonstrated the specificity and sensitivity of diiodo-squaraine PDT in cancer cells in comparison to normal breast cells (MCF-10A). However, the molecular alterations induced by diiodo-squaraine PDT in MDA-MB-231 cells have been vividly elucidated in this study. The proteomic data reveals that PDT induces oxidative stress and initiates a series of pro and anti-apoptotic pathways which include cell redox homeostasis, response to unfolded protein response, regulation of programed cell death, actin cytoskeleton organization and cell redox homeostasis in breast cancer cells. These observations were confirmed by western blots, real time PCR, confocal microscopy and flow cytometry. Based on our results, we hypothesized that localization of diiodo-squaraine in the mitochondria led to mitochondrial damage and activation of caspase cascade culminating in apoptosis. Moreover, oxidative stress induced by diiodo-squaraine in PDT disrupts the redox homeostasis which can activate the unfolded protein response and ER stress and can also trigger apoptotic cell death. Either caspase activation or oxidative stress can lead to the dynamic changes in actin cytoskeletal organization (Fig. 8). The methodology employed in this study can be adopted for investigating the molecular mechanism for other photosensitizers as well. Since the diiodo-squaraine induces predominantly apoptotic cell death pathways rather than necrosis, and lesser damage to surrounding healthy cells therefore it will be beneficial in clinical PDT. Further *in vitro* and *in vivo* studies are required to confirm this hypothesis prior to clinical trials.

## Materials and Methods

### Chemicals

Annexin V-FITC Apoptosis Detection Kit, Phalloidin–Tetramethylrhodamine B isothiocyanate and antibody to β-actin were purchased from Sigma (USA). CMH_2_DCFDA and TMRM from Life Technology, (USA), Suicide Track™ DNA Ladder Isolation Kit from Calbiochem (USA), RapiGest SF Surfactant from Waters (UK). Antibodies Chop, Hsp70, caspase3, caspase7, caspase9, catalase and PARP was purchased from Cell signaling technology (USA). Catalase and Peroxiredoxin3 were purchased from Abcam USA and secondary antibodies (HRP-conjugated anti-mouse and anti-rabbit were purchased from Santa Cruz biotechnology (USA).

### Cell culture and Photodynamic treatment

Human breast cell lines (MDA-MB-231, MCF7 and MCF 10A), colorectal cancer cells (HCT-116), cervical cancer cells (HeLa and SiHa), and pancreatic cancer cells (MIAPaCa-2) were purchased from ATCC (USA), and human oral cancer cells (SCC-131 and SCC-172) were obtained as a gift from Dr. Susanne M Gollin, University of Pittsburgh, USA, and were maintained in DMEM(Sigma, USA) containing 10% fetal bovine serum (Gibco, USA) and 1% antibiotic antimycotic cocktail (Gibco, USA). The diiodo-squaraine photosensitizer was synthesized as previously described^20^,^51^,^52^, and 100 mm stock solution in DMSO was prepared for further studies. The light source was a 700 mW–630 nM laser with optical fiber system (Vinvish LTD, India).

### Cytotoxicity assay

MTT (3-(4, 5-dimethylthiazol-2-yl)-2,5-diphenyltetrazolium bromide) reduction assay was performed to assess cell viability. Briefly, cells (10000 cells)were seeded in two 96 well cluster plate and allowed to reach the exponential phase of growth(18 hours). Later, both plates were treated using diiodo-squaraine (3.125–50 μM), incubated for 1 hour and photo-irradiated with 700 mW-630 nM laser light for 15 minutes. Another plate was kept in dark for assessing dark cytotoxicity. The amount of formazan crystals formed were measured after 4 h of MTT addition. The crystals were dissolved in isopropyl alcohol and the OD was measured at 570 nm. Graphs were plotted using Microsoft Excel, percentage inhibition were calculated by the formula (OD value of control - OD value of test)/OD value of control * 100).

### Assay for cellular reactive oxygen specious content (CM-H_2_DCFDA)

ROS stress studies, approximately 5 × 10^5^ MDA-MB-231 cells were seeded in 60 mm culture dishes and 10000 cells were plated in 60 mm and 96 well Opti-bottom plates (BD Falcon) with serum-containing media. After 18 hours, the cells were treated with 10 and 20 μM diiodo-squaraine for 1 h and photo-irradiation was done using laser 630 nM for 15 minutes. In one plate 20 μM diiodo-squaraine was added and the plate was kept in the dark to be taken as Dark control. Another plate was irradiated with a laser without drug treatment and was taken as Light control. After 1 hour of PDT the cellular reactive oxygen content was determined using a CM-H2DCFDA probe, according to the manufacturer’s instructions (Invitrogen). The confocal images and differential interference contrast (DIC) images were acquired (Nikon A1R), merged, and processed using Nikon Imaging Software. Further, we analyzed the fluorescence of CM-H_2_DCFDA using flow cytometry (FACS Aria, Special order system, BD, USA) and analysis was done using DIVA software.

### Proteomic analysis

Details of proteomic profiling and data analysis where described in Supplementary Methods.

### Immunofluorescence staining for cytoskeletal dynamics

Approximately 10000 MDA-MB-231 cells were plated in 96 well Opti-bottom plates Falcon) plates with serum-containing media. After 18 hours, the cells were treated with 10 and 20 μM diiodo-squaraine for 1 hour and photo irradiation was done using laser 630 nM for 15 minutes. In one plate 20 μM added and the plate was kept in the dark to be taken as Dark control. Another plate was irradiated with a laser without drug treatment was taken as Light control. After 24 hours. Cells were, fixed in 4% paraformaldehyde for 10 min, and then permeabilized with 0.5% Triton X 100 in PBS washed thrice in PBS, then blocked with 3%BSA in PBS for 1 hour incubated with a polyclonal anti α–tubulin antibody (1:200; Santa Cruz Biotechnology, USA) overnight. After three washes with PBS, the cells were incubated with a goat anti-mouse-FITC antibody (1:250; Santa Cruz Biotechnology, USA) for 2 h, washed thrice with PBS, and incubated with Phalloidin–Tetramethylrhodamine B isothiocyanate for 30 min and then washed with PBS twice and stained with DAPI (5 μg/ml) for 10 min. Finally, the wells were washed, and the cells were observed under a confocal microscope (Nikon A1R, Japan).

### Intracellular localization of diiodo-squaraine

For studying the intracellular localization studies 3 × 10^4^ MDA-MB-231 cells were seeded in a 8 chamber borosilicate coverless system and incubated for 24 hours. MDA-MB-231 cells were treated with 100 μM diiodo-squaraine for 1 hour and cells were fixed using 4% paraformaldehyde. The subcellular photosensitizer distribution was analyzed by confocal laser scanning microscopy. For diiodo-squaraine, we used excitation emission of 588/610 nm and cells were counterstained using nuclear stain with an excitation/emission of 405/440 nm and mitochondria using MitoTracker® Deep Red FM with an excitation/emission of 644/655 nm.

### Measure of mitochondrial membrane potential (Δψ_m_)

Mitochondrial membrane potential (Δψ_m_) was measured using TMRM dyes from molecular probes (Life Technology, USA). Approximately 5 × 10^5^ MDA-MB-231 cells were seeded in 60 mm culture dishes. After 18 hours, the cells were treated with 5, 10 and 20 μM diiodo-squaraine for 1 hour and photo irradiation was done using laser 630 nM for 15 minutes and incubated for 3,6,12 and 24 hours. Light and Dark control were taken as mentioned earlier. For fluorescence cyto-chemical study 10^5^ MDA-MB-231 cells were seeded in a 96 well plate and subjected to diiodo-squaraine PDT treatment as mentioned as earlier. Post treatment, the cells were washed with PBS and incubated with 10 nm TMRM in PBS for 30 minutes. Images were taken using a fluorescent microscope (Leica DMI 4000 B, Germany). Further, we analyzed the fluorescence of TMRM corresponding to Mitochondrial membrane potential (Δψ_m_) was analyzed using flow cytometry.

### Annexin binding assay

PDT treatment was done as mentioned as earlier in TMRM assay. Annexin binding assay was performed as per manufacturer’s instructions provided in Annexin VFITC Apoptosis Detection Kit (Sigma) and carried out flow cytometric analysis using FACS Aria.

### Western blot analysis

In Immunoblot analysis, 10^6^ MDA-MB-231 cells were seeded in 100 mm culture dishes and diiodo-squaraine PDT treatment was done as mentioned as earlier and incubated for 24 hours. Cells were lysed using RIPA buffer and quantified using Bradford’s reagent. SDS-PAGE followed by immunoblotting was carried out using 75 μg of total protein and probed using respective antibodies. Then we probed with Horseradish peroxidase(HRP)-conjugated secondary antibodies and detection was done using enhanced chemiluminescence (ECL) method. Images were captured using Molecular Imager VersaDoc MP4000 (Bio-Rad USA) system.

### RNA isolation and Real-time quantitative PCR

Approximately 5 × 10^5^ MDA-MB-231 cells were seeded in 60 mm culture. After 18 hours, the cells were treated 20 μM diiodo-squaraine for 1 hour and photo irradiation was done using laser 630 nM for 15 minutes. In one plate 20 μM added and the plate was kept in the dark to be taken as Dark control and incubated for 24 hours. Total RNA was extracted using TRIzol® Reagent (Invitrogen) manufactures instruction. Total RNA was converted cDNA into cDNA using cDNA High Capacity cDNA reverse transcription kit (Applied Bio systems). The real-time RT-PCR was performed using 7900HT real-time PCR instrument (Applied Bio systems) and the Power SYBR Green PCR master mix (Applied Biosystems). Primers designed from published sequences^24^,^53^,^54^,^55^ and qPrimerDepot a primer database^56^. Specificity of each primer was determined using NCBI BLAST module^57^. The following primers used were forward 5′-AAGGACATCAGCCAGAACAAGCG-3′ Reverse 5′-AAGAAGTCCTGCAGCAGCTTC-TGC-3′ for HSP70; forward 5′-TGTCTTTTGTCAGGGGTCTTT-3′ reverse 5′-CACAGTGGTGCCTACCAAGA-3′ for BIP; forward 5′-TCGTCCTCCGCTTTGTACTT-3′ reverse 5′-CAAGATCACCATCACCAACG-3′ for HSPA1A; forward 5′-CAG-AACCAGCAGAGGTCACA-3′ and reverse AGCTGTGCCACTTTCCTTTC-3′ for CHOP; forward 5′-TGATTACACTCCAGCGTGGTGAG-3′ and reverse 5′-CATAGATG-CCCTCTGAGACTCTGC-3′ for Catalase; and forward 5′-GTTGTCGCA-GTCTCAGTGGATTC-3′ and reverse 5′-TTCTAACAGCACACCGTAGTCTCG-3′ for PRDX3; forward 5′-GCGGAGAGGGTACAGCCAA-3′ reverse 5′-GCAGCCGGCGCAAA L19. Gene expression level of all genes was normalized with reference gene L19 and analyzed using the formula 2^−ΔΔCT^. Triplicate wells were averaged and plotted.

### DNA Ladder Assay

Approximately 5 × 10^5^ MDA-MB-231 cells were seeded in 100 mm culture dishes and diiodo-squaraine PDT treatment was done as mentioned as earlier and incubated for 24 hours. Cells were harvested, washed with PBS, and incubated with lysis buffer at 37 °C for 2−3 h. DNA was extracted with Calbiochem DNA ladder isolation kit. The precipitated DNA after centrifugation was air-dried and resuspended in Tris-HCl overnight at 55 °C. 1 μg of DNA was resolved through 2% agarose gel electrophoresis.

### Statistical analysis

All experiments were performed in triplicates. Results were normalized with respective internal controls. Data were expressed as the mean ± standard deviation (SD). ANOVA followed by Tukey’s post-hoc test was applied to compare the statistical significance of the difference between different treatment groups and control. Significance was considered at P < 0.05 and P < 0.01 was considered as highly significant.

## Additional Information

**How to cite this article**: Saneesh Babu, P. S. *et al*. Bis(3,5-diiodo-2,4,6-trihydroxyphenyl)squaraine photodynamic therapy disrupts redox homeostasis and induce mitochondria-mediated apoptosis in human breast cancer cells. *Sci. Rep.* **7**, 42126; doi: 10.1038/srep42126 (2017).

**Publisher's note:** Springer Nature remains neutral with regard to jurisdictional claims in published maps and institutional affiliations.

## Supplementary Material

Supplementary Data
