# Supplementary material for: Bis(3,5-diiodo-2,4,6-trihydroxyphenyl)squaraine photodynamic therapy disrupts redox homeostasis and induce mitochondria-mediated apoptosis in human breast cancer cells
Source: Sci Rep. 2017 Feb 7;7:42126. doi: 10.1038/srep42126 (PMC5294812; doi:10.1038/srep42126)
Supplement: Supplementary Data [file srep42126-s1.pdf]

Bis(3,5-diiodo-2,4,6-trihydroxyphenyl)squaraine photodynamic therapy disrupts redox homeostasis and induce mitochondria-mediated apoptosis in human breast cancer cells

Saneesh Babu P.S[1], Manu Prasad M[1], Dhanya T. Jayaram[3], Tapas Pradhan[1], Meera R. Nair[1], Arun Surendran[2], Aneesh Kumar A[2], Vadakkancheril S. Jisha[3], Danaboyina Ramaiah[3&4] , S. Asha Nair[1] and M. Radhakrishna Pillai[1]

[1]Rajiv Gandhi Centre for Biotechnology, Cancer Research Program, Thiruvananthapuram,695014, India. [2]Rajiv Gandhi Centre for Biotechnology ,Cardiovascular & Diabetes Disease Biology, ,Thiruvananthapuram ,695014, India. [3]CSIR-National Institute for Interdisciplinary Science and Technology , Photosciences and Photonics, Thiruvananthapuram, 695019, India. [4]CSIR-North East Institute of Science and Technology), Jorhat, 785006, India. Correspondence to S. Asha Nair (email: sasha@rgcb.res.in)

Table legends

Table S 1: List of up-regulated Proteins were identified in cell redox hemostasis(CRH) Biological process .Relative Profile shows the fold change [Ratio (Log of ratio ± Standard deviation)] observed in MDA-MB-231 Upon PDT with diiodo-squaraine (20 μM) compared to Dark control by PLGS expression analysis.

Table S 2: List of up-regulated Proteins were identified in cell response to unfolded protein (RUP)Biological process .Relative Profile shows the fold change [Ratio (Log of ratio ± Standard deviation)] observed in MDA-MB-231 Upon PDT with diiodo-squaraine (20 μM) compared to Dark control by PLGS expression analysis.

.

Table S 3: List of up-regulated Proteins were identified in regulation of actin cytoskeleton organization(ACO) Biological process .Relative Profile shows the fold change [Ratio (Log of ratio ± Standard deviation)] observed in MDA-MB-231 Upon PDT with diiodo-squaraine (20 μM) compared to Dark control by PLGS expression analysis.

Table S 4: List of up-regulated Proteins was identified in the regulation of programmed cell death(PCD) Biological Process .Relative Profile shows the fold change [Ratio (Log of ratio ± Standard deviation)] observed in MDA-MB-231 Upon PDT with diiodo-squaraine (20 μM) compared to Dark control by PLGS expression analysis

Figure legends

Figure 1: **Diiodo-diiodo-squaraine induces loss of mitochondrial membrane potential upon PDT:** Flow cytometric analysis of MDA-MB-231 cells, showing loss of MMP by diiodo-diiodo-squaraine in PDT. Data are expressed as a mean value ± standard deviation of three independent experiments. Here the population P2 shows background fluorescence, which represents cells with low MMP, and the population P3 shows cells with enhanced fluorescence indicating cells with high MMP. Here we can observe a significant decrease in P3population both at 5, 10 and 20 μM diiodo-diiodo-squaraine after 3, 6 and 12 hour of PDT as compared to both controls**.**

Figure 2: **Annexin flow cytometric analysis shows cells remain viable in early time period and sub-lethal dose of PDT**. Annexin flow cytometric analysis of MDA MB 231 cells after 3,6,12 hours of PDT treatment with light control and dark control and in presence of diiodo-diiodo-squaraine at concentration 5,10 and 20 µM. Data are expressed as a mean value ± standard deviation of three independent experiments. The lower left quadrant (Q3) of the panel shows the viable cells, negative for both Annexin V−FITC and PI. The lower right quadrants (Q4) represent the early apoptotic cells Annexin V−FITC and Propidium iodide (PI). The upper right quadrants (Q2) represent the late apoptotic cells positive for Annexin V−FITC and Propidium iodide (PI). Our results shows that cells undergoing early and late apoptosis on diiodo-diiodo-squaraine PDT was time and concentration dependent.

Table S 1

Gene Symbol	Description	PLGS Score	Relative profile	Matched Peptide
PRDX1	Peroxiredoxin-1	3502.1	2.92(0.6±0.28)	22
PRDX2	Peroxiredoxin-2	4254.3	2.48(0.91±0.22)	28
PDIA6	Protein disulfide-isomerase A6 isoform e precursor	6055.6	2.29(0.47±0.16)	27

Table S 2

Gene Symbol	Description	PLGS Score	Relative profile	Matched Peptide
CLIC1	Chloride intracellular channel protein 1	3268.25	4.31(0.76±0.81)	56
VCP	Transitional endoplasmic reticulum ATPase	5588.87	2.94(0.81±0.07)	64
HSP90AA1	Heat shock protein HSP 90-alpha isoform 2	17328.21	2.61(1.08±0.13)	23
HSP90AB1	Heat shock protein HSP 90-beta isoform c	20939.21	2.51(0.96±0.09)	18.5
HSPA8	Heat shock cognate 71 kDa protein isoform X1	30138.36	2.34(0.42±0.12)	37.5
SERPINH1	Serpin H1 isoform X1	907.67	2.14(0.55±0.25)	13

Table S 3

Gene Symbol	Description	PLGS Score	Relative Profile	Matched Peptide
RPLP2	60S acidic ribosomal protein P2	7427.57	3.82(1.34±0.34)	22
RHOA	Transforming protein RhoA precursor	3891.8	3.49(1.25±0.37)	57
PFN1	Profilin-1	48707.42	3.32(1.2±0.17)	13
EZR	Ezrin	611.34	2.86(1.05±0.55)	18
DSTN	Destrin isoform b	1645.79	2.77(1.02±0.11)	33
CFL1	Cofilin-1	11131.03	2.64(0.97±0.1)	24
CAP1	Adenylyl cyclase-associated protein 1	2369.17	2.14(0.76±0.39)	34

Table S 4

Gene Symbol	Description	PLGS Score	Relative Profile	Matched Peptide
GLO1	Lactoylglutathione lyase	725.34	5.37(1.36±0.75)	39
NME2	Nucleoside diphosphate kinase B isoform a	10750.52	3.9(1.27±0.19)	10
ARHGDIA	Rho GDP-dissociation inhibitor 1 isoform a	2631.19	3.9(1.1±0.12)	9
YWHAE	14-3-3 protein epsilon isoform X1	10173.49	3.56(1.04±0.07)	33
ACTN3	Alpha-actinin-3 isoform 1	996.73	3.56(0.68±0.08)	22
YWHAZ	14-3-3 protein zeta/delta	7797.49	3.53(0.88±0.35)	60
CFL1	Cofilin-1	11131.03	3.32(0.6±0.28)	23.5
YWHAB	14-3-3 protein beta/alpha	9167.75	3.19(1.08±0.13)	16.5
VCP	Transitional endoplasmic reticulum ATPase	5588.87	2.94(0.84±0.08)	64
PRDX1	Peroxiredoxin-1	3502.08	2.92(0.91±0.22)	22
NPM1	Nucleophosmin isoform 2	23013.82	2.83(1.2±0.17)	6
ACTN1	Alpha-actinin-1 isoform c	3476.08	2.77(0.42±0.12)	8
MIF	Macrophage migration inhibitory factor	9738.11	2.72(0.43±0.12)	5
CD44	CD44 antigen isoform 7 precursor	1361.21	2.64(0.47±0.16)	14
PRDX2	Peroxiredoxin-2	4254.29	2.48(0.46±0.1)	28
RTN4	Reticulon-4 isoform B	1183.72	2.41(0.63±0.11)	26.5
TUBB4B	Tubulin beta-4B chain	23140.22	2.32(1.26±0.14)	56.5
LGALS1	Galectin-1	11263.46	2.29(1.16±0.24)	27.5
RPL11	60S ribosomal protein L11 isoform 2	4266.17	2.03(1.07±0.24)	43

Figure SF 1

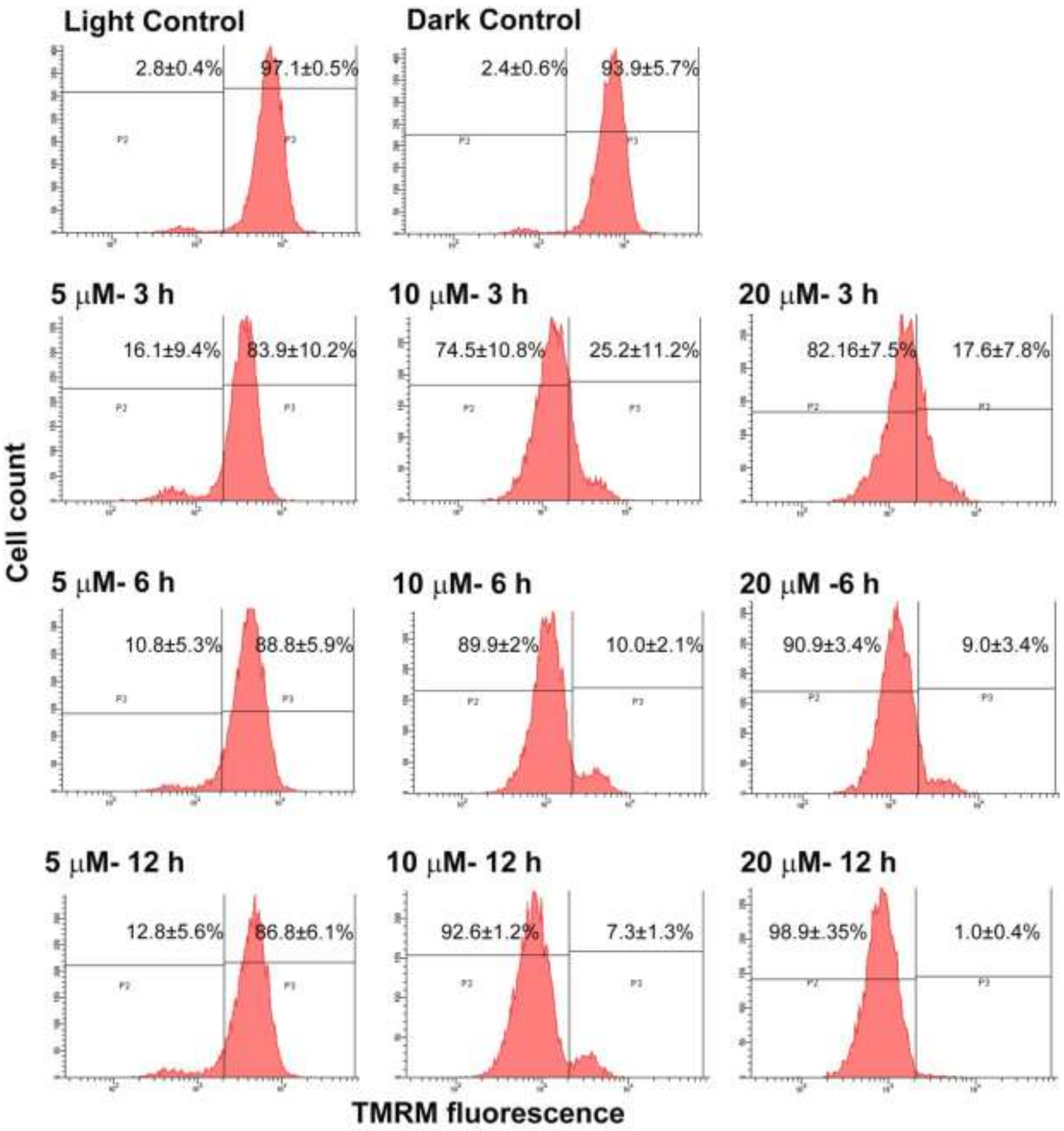

Light Control
2.8±0.4%
97.1±0.5%
Dark Control
2.4±0.6%
93.9±5.7%
5 μM- 3 h
16.1±9.4%
83.9±10.2%
10 μM- 3 h
74.5±10.8%
25.2±11.2%
20 μM- 3 h
82.16±7.5%
17.6±7.8%
5 μM- 6 h
10.8±5.3%
88.8±5.9%
10 μM- 6 h
89.9±2%
10.0±2.1%
20 μM -6 h
90.9±3.4%
9.0±3.4%
5 μM- 12 h
12.8±5.6%
86.8±6.1%
10 μM- 12 h
92.6±1.2%
7.3±1.3%
20 μM- 12 h
98.9±.35%
1.0±0.4%
Cell count
TMRM fluorescence

Figure SF 2

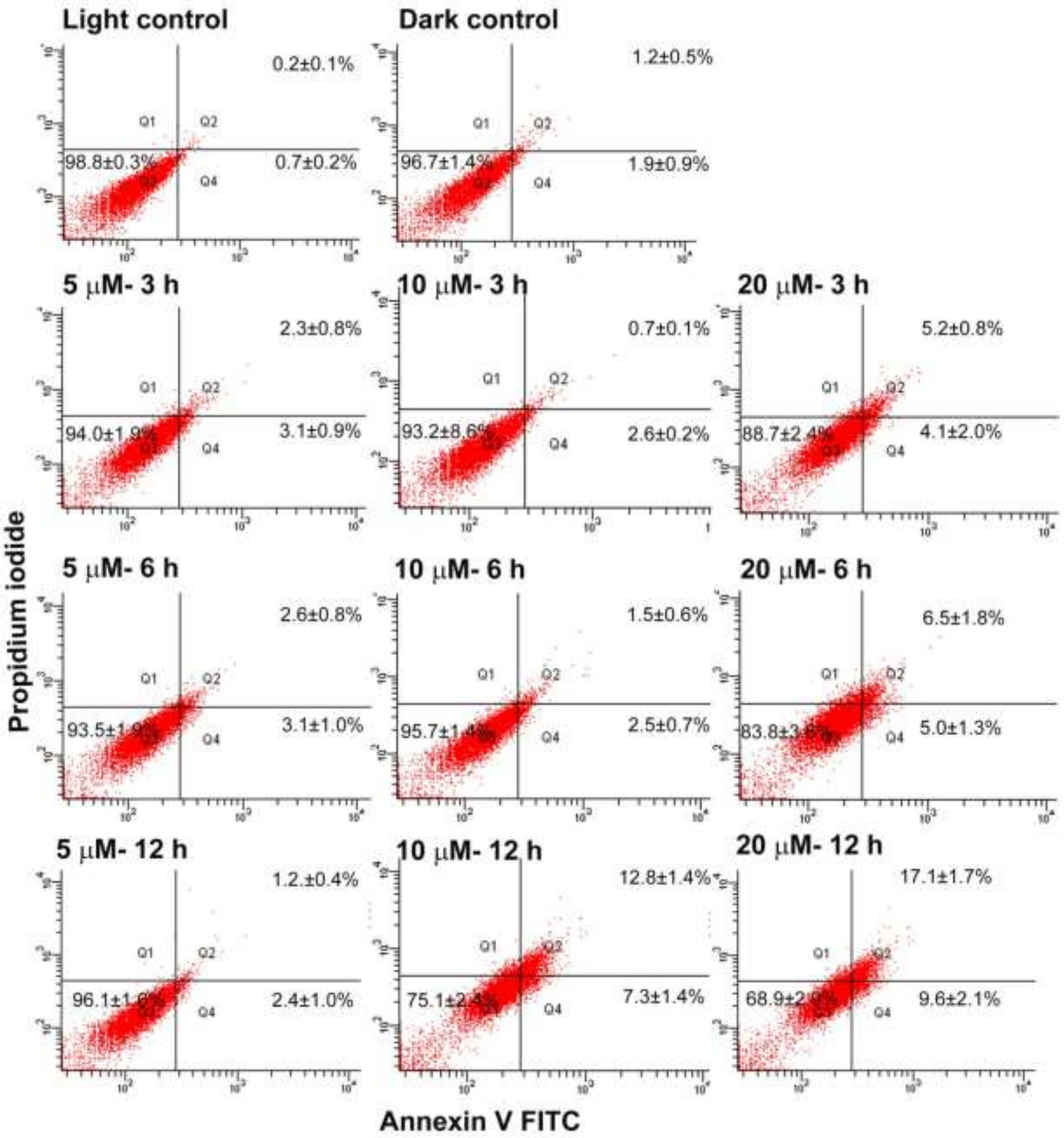
Light control
0.2±0.1%
98.8±0.3%
0.7±0.2%
Dark control
1.2±0.5%
96.7±1.4%
1.9±0.9%
5 μM- 3 h
2.3±0.8%
94.0±1.9%
3.1±0.9%
10 μM- 3 h
0.7±0.1%
93.2±8.6%
2.6±0.2%
20 μM- 3 h
5.2±0.8%
88.7±2.4%
4.1±2.0%
5 μM- 6 h
2.6±0.8%
93.5±1.9%
3.1±1.0%
10 μM- 6 h
1.5±0.6%
95.7±1.4%
2.5±0.7%
20 μM- 6 h
6.5±1.8%
83.8±3.6%
5.0±1.3%
5 μM- 12 h
1.2.±0.4%
96.1±1.6%
2.4±1.0%
10 μM- 12 h
12.8±1.4%
75.1±2.4%
7.3±1.4%
20 μM- 12 h
17.1±1.7%
68.9±2.9%
9.6±2.1%
Propidium iodide
Annexin V FITC

Supplementary Methods

Protein profiling

In this work we have used nano-flow ultra-performance liquid chromatography (nanoACQUITY UPLC® System (Waters, Manchester, UK)) coupled to a Quadrupole-Time of Flight (Q- TOF) mass spectrometer (SYNAPT-G2, Waters) for the proteomic analysis.

Sample preparation for MS analysis

Approximately 5 x 10^5 MDA-MB-231 cells were seeded in 60mm culture. After 18 h, the cells were treated 20 µM Diiodo-squaraine for 1h and photo irradiation was done using laser 630 nM for 15 minutes. In One plate 20µM added and the plate was kept in the dark to be taken as Dark control and incubated for 24 h. In another plate we did photo irradiation without any compound and taken as our light control . Cell lysis and protein extraction of the sample were done with 0.3% w/v of RapiGest SF surfactant (Waters) in 50 mM ammonium bicarbonate (ABC). The samples were centrifuged at 14,000 rpm for 15 min and protein concentration of the supernatant was measured using Bradford assay. The concentrations of the sample were normalized by adding ABC to yield a final concentration of 1 µg/µL. Approximately 100 µg of protein from each sample was subjected to in-solution trypsin digestion to generate peptides. The samples were heated at 80°C while shaking for 15 min to increase protein solubility. Disulfide bonds were reduced by incubation of proteins with 100 mM dithiothreitol (DTT) in ABC for 30 min at 60ºC. After cooling at room temperature for 5 min, the samples were spun and 200 mM iodoacetamide (IAA) in ABC was added to perform the alkylation step in the dark at room temperature for 30 min. Proteins were then digested by using sequencing grade modified trypsin (Sigma) at a trypsin: total protein ratio of ~1:25 and incubated for 17 h at 37ºC. The trypsin stock was made in ABC. The reaction was stopped by adding

1 µL of 100% formic acid and incubating at 37°C for 20 min. The digested peptide solutions were centrifuged at 14,000 rpm for 12 min and the supernatant was collected. MassPREP™ Digestion Standard (MPDS, Waters), a set of tryptic digested peptides of four proteins were spiked in different ratios between the peptide samples for the eventual protein expression analysis by label-free method. MPDS consists of 4 proteins, namely Bovine Serum Albumin (BSA), Rabbit Glycogen Phosphorylase b (GPB), Yeast Alcohol Dehydrogenase (ADH), and Yeast Enolase I (ENO). This MPDS were available as Protein Expression Mixture-1(MPDS-1) and Protein Expression Mixture-2 (MPDS-2) having 1:2:8:0.5 fold abundance of peptides for ADH, ENO, BSA, and GPB respectively in MPDS-2. Either MPDS-1or MPDS-2 was spiked with each of the samples as internal control for protein expression. The peptide samples spiked with MPDS were transferred to auto sampler vials (Total Recovery Vial, Waters) for peptide analysis via LC-MS^{E} (MS at elevated energy) with ion-mobility.

Separation by nanoflow liquid chromatography

The tryptic peptides were separated using a nanoACQUITY UPLC® chromatography system (Waters, Manchester, UK). Instrument control and data processing was done with MassLynx4.1 SCN781 software. In the nanoACQUITY UPLC®, the peptides were separated by reversed-phase chromatography technology. The peptide sample was injected in the partial loop mode in 5 µL loop (injection volume 3.0 µL) .Water was used as mobile phase A and acetonitrile was used as a mobile phase B. All mobile phases in the UPLC system contained 0.1 % formic acid. The tryptic peptides were trapped and desalted on a trap column (Symmetry® 180µm x 20mm C18 5µm, Waters) for 1 minute with a flow rate of 15 µL/min. The trap column was placed in line with the reversed-phase analytical column, a 75 µm i.d. X 200 mm BEH C18 (Waters) with particle size of 1.7 µm Peptides were eluted from the column with a linear gradient of 1 to 40% mobile phase B over 55.5 min at a flow rate of 300 nL/min followed by a 7.5 min rinse of 80 % mobile phase B. The column was immediately re-equilibrated at initial conditions (1% mobile phase B) for 20 min. The column temperature was maintained at 40° C. The *lock mass*, [Glu^{1}]-Fibrinopeptide B human (Sigma) was delivered from the auxiliary pump of the UPLC system through the reference sprayer of the NanoLockSpray™source at a

flow rate of 500 nL/min. Each sample was injected in triplicate with blank injections between each sample.

MS analysis

Mass spectral analysis of eluting peptides from the nanoACQUITY UPLC® was carried out on a SYNAPT® G2 High Definition MS™ System (HDMS^E System (Waters). It is a hybrid, Quadrupole, ion mobility, orthogonal acceleration, time-of-flight mass spectrometer controlled by MassLynx4.1 SCN781 (Waters Corporation, Milford, MA, USA) software. Integrating mass spectrometry with ion mobility enables an extra dimension of sample separation and definition by combining exact-mass to charge, intensity, and drift time. The instrument settings include: nano-ESI capillary voltage – 3.4 kV, sample cone - 40 V, extraction cone - 4 V; trap gas flow - 14 (mL/min), IMS gas (N_2) flow - 90 (mL/min). To perform the mobility separation, the IMS T-Wave™ pulse height is set to 40 V during transmission and the IMS T-Wave™ velocity was set to 800 m/s. The travelling wave height was ramped over 100% of the IMS cycle between 8 V and 20 V.

All analyses were performed using positive mode ESI using a NanoLockSpray™source. The lock mass channel was sampled every 45 s. The time of flight analyzer (TOF) of the mass spectrometer was calibrated with a solution of 500 fmole/µL of [Glu^1]-Fibrinopeptide B human (Sigma). This calibration sets the analyzer detectionsons in the range of 50 - 2,000 *m/z*. The mass spectrometer was operated in resolution mode (V mode) with a resolving power of 18,000 FWHM and the data acquisition was done in *continuum* format. The data were acquired by rapidly alternating between two functions – Function-1 (low energy) and Function-2 (high energy). In Function-1, we acquire only low energy, mass spectra (MS) and in Function-2, we acquire mass spectra at the elevated collision energy with ion mobility (HDMS^E). InFunction-1, collision energy was set to 4 eV in the Trap region and 2 eV in the Transfer region. In Function-2, collision energy was set to 4 eV in the Trap region and is ramped from 20 eV to 45 eV in the

Transfer region to attain fragmentation in the $HDMS^E$ mode. The *continuum* spectral acquisition time in each function was 0.9 seconds with an inter can delay of 0.024s.

Data processing, protein identification and network analysis

The *continuum* $HDMS^E$ data were analyzed using ProteinLynx Global SERVER™ v2.5.3 (PLGS, Waters) for protein identification as well as for the label-free relative protein quantification. Processing parameters for PLGS were set as follows: noise reduction thresholds for low energy scan ion – 150 counts, high energy scan ion - 50 counts and peptide intensity - 500 counts (as suggested by manufacturer). The protein identifications were obtained by searching against the human database (retrieved from NCBI on June 2014, with 71340 sequences) to which data of the MPDS were appended. During database search, the protein *false positive rate* was set to 4 %. The parameters for protein identification was made in such a way that a peptide was required to have at least 1 fragment ion match, a protein was required to have at least 3 fragment ion matches and a protein was required to have at least 1 peptide match for identification. *Oxidation* of methionine was selected as variable modification and cysteine *carbamidomethylation* was selected as a fixed modification. Trypsin was chosen as the enzyme used with a specificity of 1 missed cleavage. Data sets were normalized using the 'internal standard-normalization' function of PLGS and label-free quantitative analyses were performed by comparing the normalized peak area/intensity of identified peptides between the samples. ADH was chosen as the internal standard for normalized during relative protein quantification. Thus, we obtained parameters such as score, sequence coverage and number of peptides identified for each protein. The protein data set was filtered by considering only those identified proteins which have at least 2 peptides. Furthermore, only a fold change higher than two fold difference in 20 μM (diiodo-squaraine PDT) when compared to dark control (ratio of >2) was considered to be indicative of significantly altered levels of expression. We subtracted those protein significantly overexpressed in light control compared to dark control for eliminate the effect of photo irradiation in this experiment. Reference sequence

protein accession was converted into Gene ID *via* BioDB net [1]. Enriched gene ontology (GO) was identified using DAVID Bioinformatics Resources 6.7[2]. A Venn diagram was drawn using proteins in different Bioprocess (BP) [3]. Network Assembly of Functional Protein Interactions observed from Bioprocess obtained from DAVID were submitted to the web based STRING database [4,5] to generate functional interaction networks based on their negative logarithm of P value (higher significance).

.

1 Mudunuri, U., Che, A., Yi, M. & Stephens, R. M. bioDBnet: the biological database network. *Bioinformatics* **25**, 555-556 (2009).

2 Huang, D. W., Sherman, B. T. & Lempicki, R. A. Systematic and integrative analysis of large gene lists using DAVID bioinformatics resources. *Nature protocols* **4**, 44-57 (2008).

3 Oliveros, J. C. VENNY. An interactive tool for comparing lists with Venn Diagrams. (2007).

4 Szklarczyk, D. *et al.* The STRING database in 2011: functional interaction networks of proteins, globally integrated and scored. *Nucleic acids research* **39**, D561-D568 (2011).

5 Diner, B. A., Lum, K. K., Javitt, A. & Cristea, I. M. Interactions of the Antiviral Factor Interferon Gamma-Inducible Protein 16 (IFI16) Mediate Immune Signaling and Herpes Simplex Virus-1 Immunosuppression. *Molecular & Cellular Proteomics* **14**, 2341-2356 (2015).
